# Positive selection and precipitation effects on the mitochondrial *NADH dehydrogenase subunit 6* gene in brown hares (*Lepus europaeus*) under a phylogeographic perspective

**DOI:** 10.1371/journal.pone.0224902

**Published:** 2019-11-08

**Authors:** Milomir Stefanović, Mihajla Djan, Nevena Veličković, Dejan Beuković, Vukan Lavadinović, Chavdar Dinev Zhelev, Yasin Demirbaş, Ladislav Paule, Csongor István Gedeon, Zissis Mamuris, Annika Posautz, Christoph Beiglböck, Anna Kübber-Heiss, Franz Suchentrunk

**Affiliations:** 1 Department of Biology and Ecology, Faculty of Sciences, University of Novi Sad, Novi Sad, Serbia; 2 Faculty of Agriculture, University of Novi Sad, Novi Sad, Serbia; 3 Faculty of Forestry, University of Belgrade, Belgrade, Serbia; 4 Southwestern State Forest Enterprise Blagoevgrad, Blagoevgrad, Bulgaria; 5 Faculty of Science and Arts, University of Kırıkkale, Kırıkkale, Turkey; 6 Faculty of Forestry, Technical University, Zvolen, Slovakia; 7 Institute for Soil Sciences and Agricultural Chemistry, Hungarian Academy of Sciences, Budapest, Hungary; 8 Department of Biochemistry and Biotechnology, University of Thessaly, Larrisa, Greece; 9 Research Institute of Wildlife Ecology, University of Veterinary Medicine Vienna, Vienna, Austria; Banaras Hindu University, INDIA

## Abstract

Previous studies in hares and jackrabbits have indicated that positive selection has shaped the genetic diversity of mitochondrial genes involved in oxidative phosphorylation, which may affect cellular energy production and cause regional adaptation to different environmental (climatic) pressures. In the present study, we sequenced the *NADH dehydrogenase subunit 6* (*MT-ND6*) gene of 267 brown hares (*L*. *europaeus*) from Europe and Asia Minor and tested for positive selection and adaptations acting on amino acid sequences (protein variants). Molecular diversity indices and spatial clustering were assessed by DnaSP, Network, and Geneland, while the presence of selection signals was tested by codeml in PAML, and by using the Datamonkey Adaptive Evolution web server. The SPSS software was used to run multinomial regression models to test for possible effects of climate parameters on the currently obtained protein variants. Fifty-eight haplotypes were revealed with a haplotype diversity of 0.817, coding for 17 different protein variants. The *MT-ND6* phylogeographic pattern as determined by the nucleotide sequences followed the earlier found model based on the neutrally evolving D-loop sequences, and reflected the earlier found phylogeographic Late Pleistocene scenario. Based on several selection tests, only one codon position consistently proved to be under positive selection. It did occur exclusively in the evolutionarily younger hares from Europe and it gave rise to several protein variants from the southeastern and south-central Balkans. The occurrence of several of those variants was significantly favored under certain precipitation conditions, as proved by our multinomial regression models. Possibly, the great altitudinal variation in the Balkans may have lead to bigger changes in precipitation across that region and this may have imposed an evolutionarily novel selective pressure on the protein variants and could have led to regional adaptation.

## Introduction

Mitochondria play a key role in cellular energy and heat production of organisms. The mitochondrial genome contains thirteen protein-coding genes, which along with a number of nuclear genes encode the protein subunits that make up four out of five complexes of the electron transport chain (ETC) where the oxidative phosphorylation (OXPHOS) pathway occurs [1]. Mitochondrial DNA (mtDNA) is widely used as a marker for phylogenetics, phylogeography, and population genetics studies, because of its high mutation rate, maternal inheritance, little or no recombination, its compact strcutre of coding DNA, as well as the assumption to evolve under neutral or nearly-neutral expectations [2–3]. However, given the functional importance of some of the peptides encoded by mtDNA, and the likely fitness implications of peptide variants, selection on mitochondrial genes may be influenced by environmental conditions affecting metabolic processes [4]. Information on the evidence of positive selection acting on the molecular level and on adaptive signals in mtDNA have been provided in a wide range of taxa from *Drosophila* to humans [5–11].

The mitochondrially encoded *NADH dehydrogenase* subunit 6 gene (*MT-ND6*) encodes one of the seven hydrophobic subunits of the OXPHOS Complex I and is located in the cysteine-rich inner light strand [12]. Apart from the D-loop that is usually the most polymorphic part of the mtDNA, *MT-ND6* is one of the most polymorphic mitochondrial structural genes in vertebrates [13]. This gene encodes the ND6 subunit that seems to be important for the assembly of Complex I [14]; in humans various point mutations of the *MT-ND6* gene affect NADH dehydrogenase activity [15] and are associated with diseases such as Leber’s optic neuropathy, encephalomyopathy, and stroke-like episodes [16]. Moreover, certain positions of the *MT-ND6* gene seem to be important in physiological adaptations to high altitude regions, as reported for snub-nosed monkey [17], alpine pheasants [9], and Tibetan horses [18].

Hares and jackrabbits (*Lepus spp*.) within the order Lagomorpha represent a highly polymorphic group of closely related mammal species widely distributed in a range of contrasting environments. In a study of complete mitochondrial genomes of eleven *Lepus* species, several codons in the OXPHOS complexes showed evidence of positive selection, suggesting adaptations affecting the evolution of hare mtDNA [10]. Correspondingly, Ben Slimen et al. [6] revealed positive selection in the *ATP synthase 6* (*MT-ATP6*) and *NADH dehydrogenase 2* (*MT-ND2*) genes across 22 *Lepus* species, as well as significant climate effects on the geographical distribution of several translated protein variants. Furthermore, a study of cape hares (*Lepus capensis* sensu lato) from a steep ecological gradient in northern Africa strongly suggested a role of ambient temperature in the adaptation of the *MT-ATP6* and *MT-ND2* genes [7].

Brown hares (*Lepus europaeus*) are widespread throughout Europe and Asia Minor and other parts of western and southwestern Eurasia, occurring in a variety of environments with a considerable ecological plasticity (see e.g., [19]). MtDNA-based phylogeographic and ancient demographic data indicate distinct regional evolutionary units for this species [20–25], despite relatively little changes (i.e., high gene flow) across larger geographical distances in the nuclear genome [26–35]. According to the phylogeographic model based on non-coding D-loop sequences of the mtDNA proposed by Djan et al. [21], the southeastern central Balkans has been identified as the source of all populations of the southern and northern Balkans, while populations from central and northwestern Europe have originated from the northern Balkans, and all Balkan lineages have been derived from evolutionarily older lineages from the Anatolian Peninsula. Additional routes of hares colonizing eastern and northeastern Europe via west or east of the Black Sea are suggested by Strzala et al. [36] and Ashrafzadeh et al [20]. As the mtDNA genetically represents a haploid, maternally inherited linkage group with a high mutation rate, with no or very little recombination, it can be expected that genetic differentiation in other mtDNA genes, such as *MT-ND6*, will follow the same signal of phylogeographic partitioning as observed for D-loop lineages. Demographic history analyses of hares from Europe and Asia Minor indicated a longer evolutionary period in Anatolia than in Europe [21]. Because of that and the phylogeographic scenario stated above, higher genetic diversity may be expected in the Anatolian Peninsula than in central Europe and possibly also in southeastern Europe, i.e., in the Balkans.

Here, we study the geographic distribution of *MT-ND6* haplotypes in brown hares from the Middle East and Europe, in particular from Anatolia, Israel, the Balkans, central, and northwestern-central Europe that were demonstrated earlier to share a common evolutionary history, and the potential causes of their distribution. In principle, two major evolutionary processes may have shaped the distributional pattern of haplotypes across the study area: firstly, purely neutral evolutionary processes, such as gene flow and regional random drift due to various potential (unknown) population dynamic changes in the evolutionary history, in accordance with the earlier observed distribution of D-loop haplotypes; and secondly, negative or/and positive selection, potentially having only regionally led to deviations from the distributional pattern as expected from neutral evolution. Following Ben Slimen et al. [6–7] and Melo-Ferreira et al. [10] that have demonstrated positive selection in mtDNA of various species of hares and jackrabbits (genus *Lepus*) and accounting for the fact that isolation of populations in distinct (Pleistocene) refugia may have led to adaptations and differentiation in allopatry [37], we hypothesize that molecular diversity at the particularly highly variable *MT-ND6* locus has been shaped by positive selection and adaptation to varying environments in the course of its phylogenetic history. Specifically, positive selection may have led to adaptation in populations from Europe, where more pronounced climatic oscillations have occurred during the Late Pleistocene, with massive ice cover shifts and concomitant extreme changes of vegetation on north-south and east west gradients [38]. Hence, we specifically expect positive selection and adaptation signals in hares from Europe, whereas positions under negative selection may occur throughout our entire study range. However, as indicated by the phylogeographic mtDNA model of Djan et al. [21] (see also [24]), the postglacial colonization of central Europe by brown hares has exclusively started from the northern Balkan populations and was based on only few haplotypes, the evolutionary time span might have been too short for generating an high enough number of protein variants amongst central European hares to register adaptation signals. The model of ancient population dynamics on the Balkans, however, together with the comparatively high level of mtDNA D-loop sequences, may have lead already to sufficient diversity at the *MT-ND6* locus, allowing adaptation to varying environmental conditions. Conversely, the evolutionarily more ancestral brown hares from the Middle East (i.e., the Anatolian Peninsula and Israel studied currently) may have experienced enough chances for adaptation, compared to the somewhat shorter evolutionary time in Europe. As some typical Anatolian/Middle Eastern mtDNA lineages have reached the southeastern Balkans [21, 22, 24], we may expect adaptations in brown hares from southeastern Europe that carry particularly protein variants that have evolved from Anatolian/Middle Eastern ancestor proteins. To test these hypotheses we developed a phylogeographic model of *MT-ND6* sequences, tested for positive selection using codon-based approaches, and searched for adaptive signals by testing for significant climate effects on the geographic distribution of protein variants.

## Material and methods

### Sequencing of the *MT-ND6* gene

**Fig 1:**
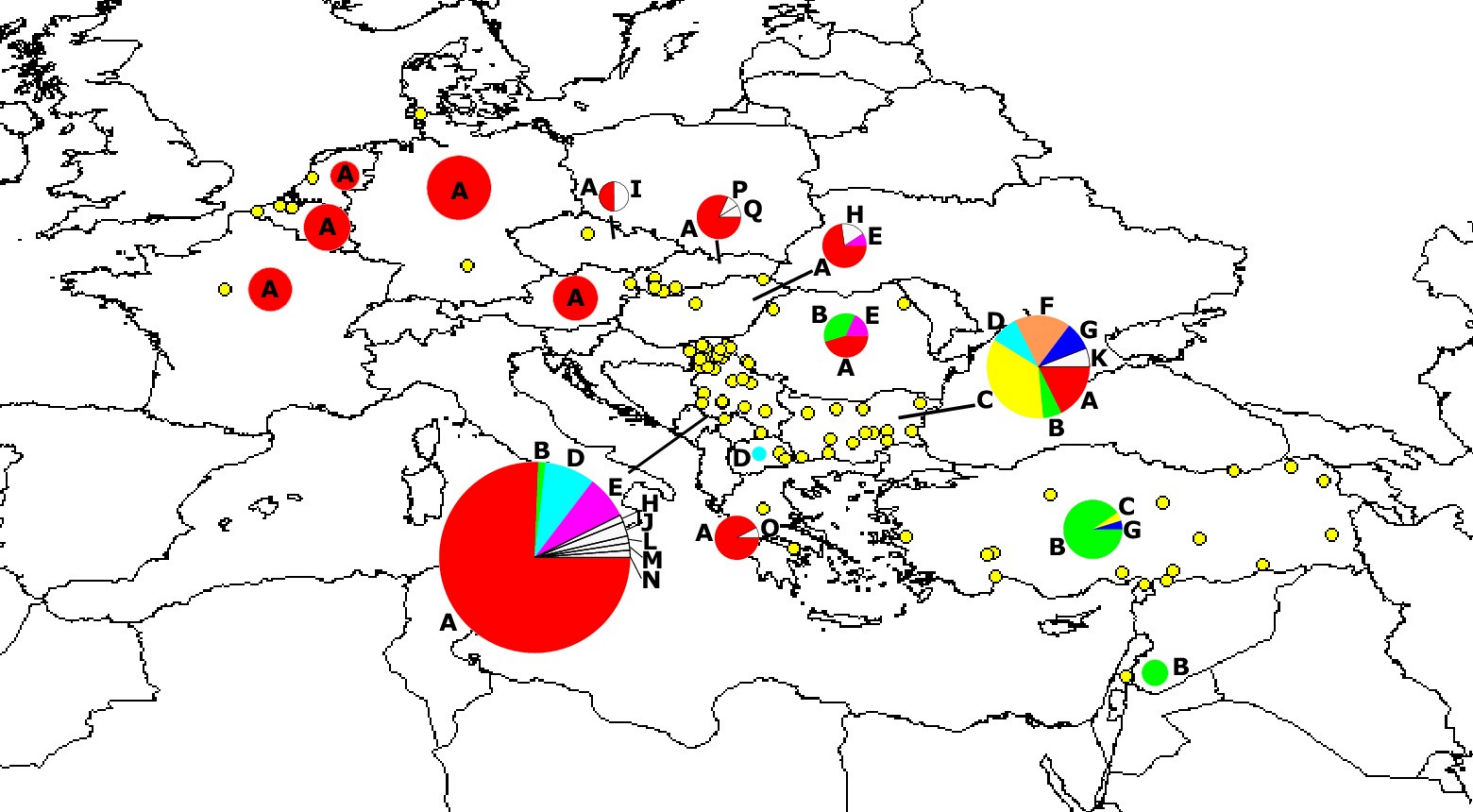
Geographic positions of sampling localities of *L*. *europaeus* (yellow circles) from Europe and the Middle East. Pie charts represent the frequency distribution of *MT-ND6* protein variants by countries, with pie sizes corresponding to country-wide sample sizes. All protein variants with country wide frequencies below 3% are indicated as white slices, together with the corresponding letter of protein designation.

Skeletal muscle tissue samples of 267 brown hares (*Lepus europaeus* Pallas, 1778) from various localities (Fig 1, Table 1) across central Europe, the Balkans, the Anatolian Peninsula, and Israel were collected during regular hunts and stored at -20°C until laboratory work. Animals were not shot for the purpose of this study. Samples were collected from hunters following the regulations in brown hare management. An ethics statement was not required. Genomic DNA was extracted using a slightly modified protocol as published by [39]. The entire coding sequence of the mitochondrially encoded *NADH Dehydrogenase* Subunit 6 gene (*MT-ND6*) was amplified with the following primer pair designed for this study: forward primer 5'CAATACACCGCCTCTTACCT3' and reverse primer 5'GGTGCGTTTTACGAATGTTG3'. Approximately 100 ng of genomic DNA were amplified in a total volume of 25 μl containing 0.2 mM dNTP, 0.2 μM of each primer, 1U Dream Taq DNA polymerase (Thermo Fischer Scientific, Waltham, Massachusetts, United States) and 1X Dream Taq reaction buffer (containing 20 mM MgCl_2_). PCR amplification conditions were as follows: initial step of denaturation at 95°C for 4 min, followed by 30 cycles of amplification–each cycle being 95°C for 60s, 54°C for 45s, and 72°C for 45s –and a final extension step at 72°C for 5 min. The PCR products were purified following the Exo SAP protocol (Thermo Fischer Scientific, Waltham, Massachusetts, United States) and sequencing was conducted using the BigDye Terminator Cycle Sequencing kit on an ABI 3130xl DNA Analyzer (Applied Biosystems, Foster City, California, USA). Sequences were aligned using the Clustal algorithm implemented in BioEdit 7.0.9.0. [40], with final adjustments by eye.

**Table 1:** Distribution of haplotypes and protein variants of *MT-ND6* in brown hares from Europe and the Middle East (for Serbia two regions were distinguished to account for marked phylogeographic differentiation, see [21]).

Country	Sample size	*MT-ND6* Haplotypes	*MT-ND6* protein variants
Austria	13	H1	A
Belgium	14	H1, H2	A
Bulgaria	34	H1, H3-H9, H56-H58	A, B, C, D, F, G, K
Czech Republic	5	H1, H10	A, I
France	11	H1, H11	A
North Macedonia	2	H12	D
Germany	26	H1	A
Greece	13	H13, H14	A, O
Hungary	11	H1, H16-H18	A, E, H
Israel	7	H19, H20	B
Netherlands	6	H1, H21	A
Romania	11	H1, H16, H22-H26	A, B, E
Serbia	82		
*Vojvodina*		H1, H16-H18, H23, H29, H33, H35, H36	A, B, D, E, H, N
*Central Serbia*		H1, H9, H13, H18, H25, H27, H28-H32, H34	A, D, E, J, L, M
Slovakia	11	H1, H37, H38, H39	A, P, Q
Turkey	21	H40 –H55	B, C, G

### Molecular diversity

Overall molecular diversity indices (h–number of haplotypes, Hd–haplotype diversity, π –nucleotide diversity, k–mean number of pairwise differences) were calculated using DnaSP 5.10 [41]. Neutrality tests (Fu’s Fs and Tajima’s D tests), as well as mismatch analyses were performed in ARLEQUIN 3.5.1.2 [42], while DnaSP was used to run Fu and Li F* and D* tests, based on estimations of segregating sites.

### Phylogenetics and spatial clustering of *MT-ND6* genes

In order to assess phylogenetic relations between the revealed haplotypes a median-joining (MJ) network [43] was constructed with the software Network 4.6.0.0 (available at http://www.fluxus-engineering.com/sharenet.htm). All positions were equally weighted and the ε parameter was set to zero. The MJ network was rooted using haplotypes of a cape hare, *Lepus capensis* s. l. (Gen Bank accession number: NC_015841), a mountain hare, *L*. *timidus* (NC_024040) and an Iberian hare, *L*. *granatensis* (NC_024042) as out-groups. Additionally, mismatch distribution and neutrality tests were performed in ARLEQUIN 3.5.1.2. Spatial clustering of individual *MT-ND6* sequences was performed using Geneland 3.0 [44]. The uncorrelated model based on a multinomial distribution of genotypes conditionally based on allele frequencies, population memberships, and linkage equilibrium was used with a total of 1.000.000 iterations in 10 independent runs and the number of clusters (k) from 1 to 10, with sampling every 100 steps and discarding the first 30% as burn-in. Three additional runs were performed using the same parameters with selected fixed K value. However, the phylogeographic analyses should account for evolutionary processes over temporal, as well as spatial scale, which cannot be provided by the above mentioned approaches simultaneously. Therefore, the phylogeographic partitioning of the *MT-ND6* sequences was tested by a coalescent-based model as implemented in the R package BPEC (Bayesian Phylogeographic and Ecological Clustering; [45]), which accounts for haplotype connection ambiguities due to the loops and gives the estimates of posterior probabilities over the suggested clustering. Two MCMC chains were run for 20 million iterations with sampling every 1000 step. For each run, a strict parsimony criterion was applied and the maximum number of migrations was 5. Molecular DNA polymorphism indices, neutrality tests, and mismatch distribution analysis were also calculated for each detected spatial cluster using DnaSP and ARLEQUIN. Genetic differentiation within and between the detected clusters was further assessed by calculating Φ_ST_ pairwise differences and through an analysis of molecular variance (AMOVA) using ARLEQUIN.

### Selection analyses

The effect of natural selection acting on the evolution of the *MT-ND6* gene was assessed by maximum likelihood and Bayesian approaches, comparing the number of nonsynonymous changes per nonsynonymous sites to the number of synonymous changes per synonymous site. The “individual codon site model”implemented in codeml in the PAML package [46], was used for comparisons, each involving a null model (M0, M1a, M7) and a positive selection model (M3, M2a, M8). Specifically, we compared models M0 vs. M3, M1a vs. M2a and M7 vs. M8. Statistically significant evidence of positive selection was inferred by a likelihood ratio test (LRT) comparing 2× the log likelihood difference of each set of nested models. These values were compared to the χ2 distribution with the appropriate degrees of freedom. Additional codon models (SLAC—Single Likelihood Ancestral Counting; FEL–Fixed Effects Likelihood; REL–Random Effects Likelihood; MEME—Mixed Effects Model of Evolution) as implemented on the Datamonkey web server (http://www.datamonkey.org/, last accessed September 2018) were used, based on a GARD inferred tree. Sites with cut-off values of p<0.1 in SLAC, FEL, and MEME and Bayes factors > 50 in REL were considered as candidates to have evolved under positive selection. In order to predict functional relevance of amino-acids proven to be under positive selection we applied several algorithms for protein domain architecture and secondary structure predictions, such as TMHMM [47], I-TASSER [48], PSI-pred [49] and PHYRE threading program (http://www.sbg.bio.ic.ac.uk/~phyre/). Since even the homologous amino-acid sequences from the same protein family can have inverted topologies, we have used all the amino-acid variants detected in this study as templates for all of the mentioned software, and considered the final predicted secondary structure model as an overall integration of each algorithm results.

### Adaptation analysis

Given that previous research in hares [6–7] has reported climatic effects on the evolution of sequences encoding for mt OXPHOS genes, we used SPSS vers.20 (IBM SPSS Statistics for Windows, Version 20.0. Armonk, NY: IBM Corp) to run multinomial regression models to test for possible effects of climate parameters on the currently obtained *MT-ND6* protein variants. However, prior to those model runs we performed two principal component analyses (PCA) for the “world bioclim data”downloaded for each sampling locality from the http://www.worldclim.org/bioclim platform. In particular, one PCA focused on reducing the number of all temperature related bioclimatic data (i.e., bio 1, 5, 6, 8, 9, 10, 11) and the second on reducing the precipitation related bioclimatic data (i.e., bio 12–19, except bio 15) to obtain meaningful climatological factors. Prior to PCA, all climate variables were *ln*-transformed to reduce variances and to obtain normal distributions of residuals. After checks for possible multicollinearity in the data, multinomial regression modeling was performed on the occurrence of the most prevalent protein variants as dependent variable and individual principal component factor scores resulting from the bioclimatic variables, controlling at the same time for likely geographic (latitude, longitude, altitude) variables.

## Results

**Fig 2:**
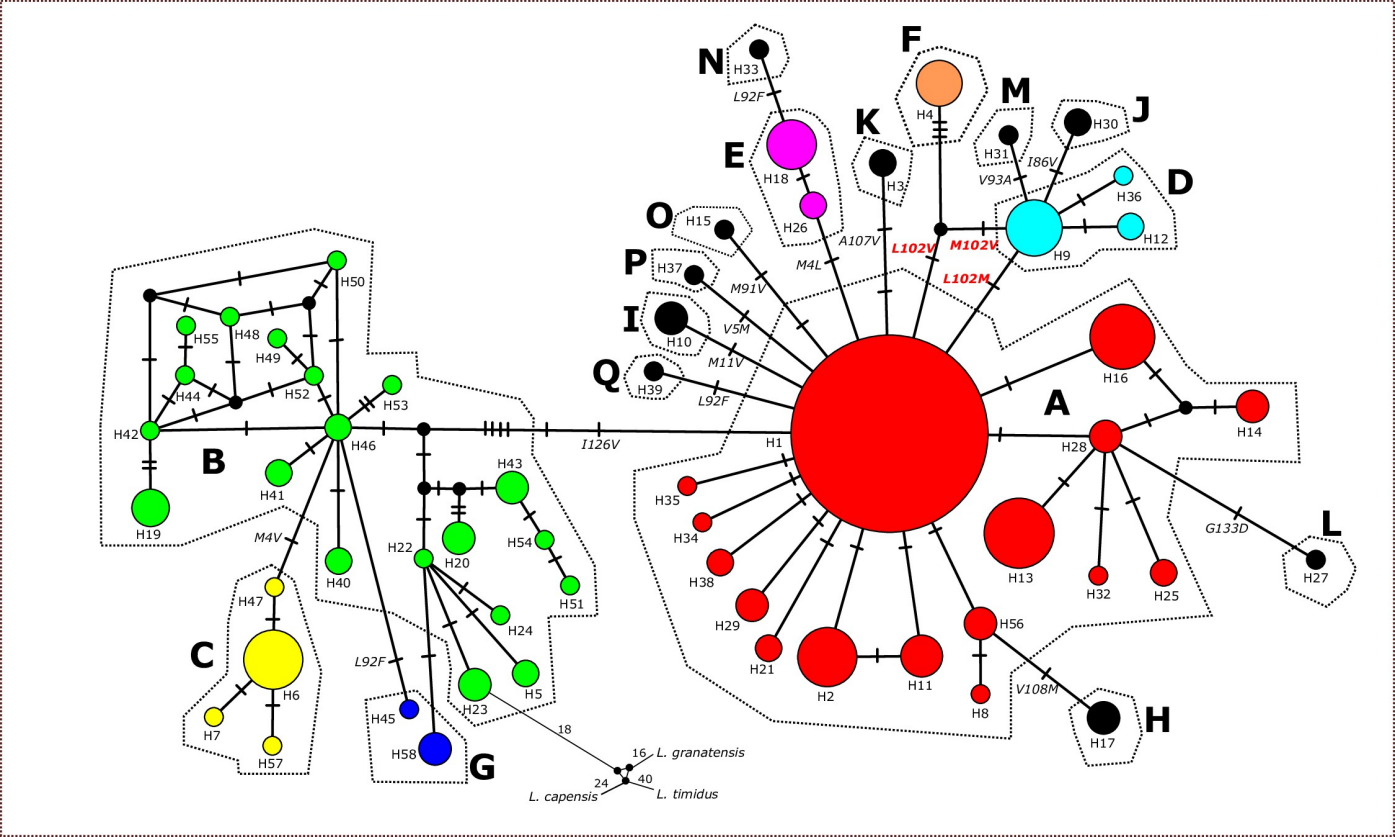
Median-joining network showing the evolutionary relationships among *MT-ND6* haplotypes. Relative haplotype frequencies correspond to haplotype circle size, while the number of perpendicular bars on lines connecting any two haplotypes corresponds to the number of mutation changes. Localities of each haplotype correspond to those as given in Table 1. Dashed lines encircle different amino-acid sequences (protein variants) coded as A to Q; the position 102 indicates the position that was proved under positive selection by more than two of our tests (details see Material and Methods, and Results sections).

Among all 267 *MT-ND6* sequences of brown hares from Europe and the Middle East (Anatolia and Israel) obtained in the current study, 58 different haplotypes were detected (GenBank accession numbers MN124689-MN124746), based on 56 polymorphic sites of which 40 were parsimony informative and 16 were singletons. Haplotype diversity (Hd) amounted to 0.817±0.024, nucleotide diversity (π) to 0.008, and the average number of nucleotide differences (k) to 4.055. The majority of haplotypes was represented by low frequencies ranging from 0.37% to 5.24%, with 27 unique haplotypes. The most common haplotype was H1, occurring in 41.57% of all analyzed individuals (Table 1, Fig 2).

Our MJ network model of phylogenetic relationships between haplotypes revealed two phylogroups, the “Anatolian/Middle Eastern”(AM), and the “European (EU)”phylogroup. Within the AM phylogroup, 25 haplotypes were revealed with Hd = 0.943, based on 24 polymorphic sites, whearas the EU phylogroup consisted of 33 haplotypes with Hd = 0.729 based on 39 polymorphic sites. Nucleotide diversity (π) was significantly higher in the AM phylogroup (π = 0.008±0.00044) than in the EU phylogroup (π = 0.003±0.00028) because the upper bound of the double s.d. of π for the EU phylogroup was well below the lower bound of the double s.d. of π for the AM phylogroup (duality principle in test statistics). Similarly, k values were higher in the AM phylogroup (k = 4.408) than in the EU phylogroup (k = 1.574). Rooting the network by the three outgroup taxa indicated an ancestral evolutionary status of the AM phylogroup and a derived status of the EU phylogroup. This was in accordance with the star-like pattern of haplotype relationships, the significantly negative Tajima´s D (-2.193; p < 0.01) and Fu´s Fs (-27.94; p < 0.01) values that suggested a recent rapid radiation for the EU phylogroup.

**Fig 3:**
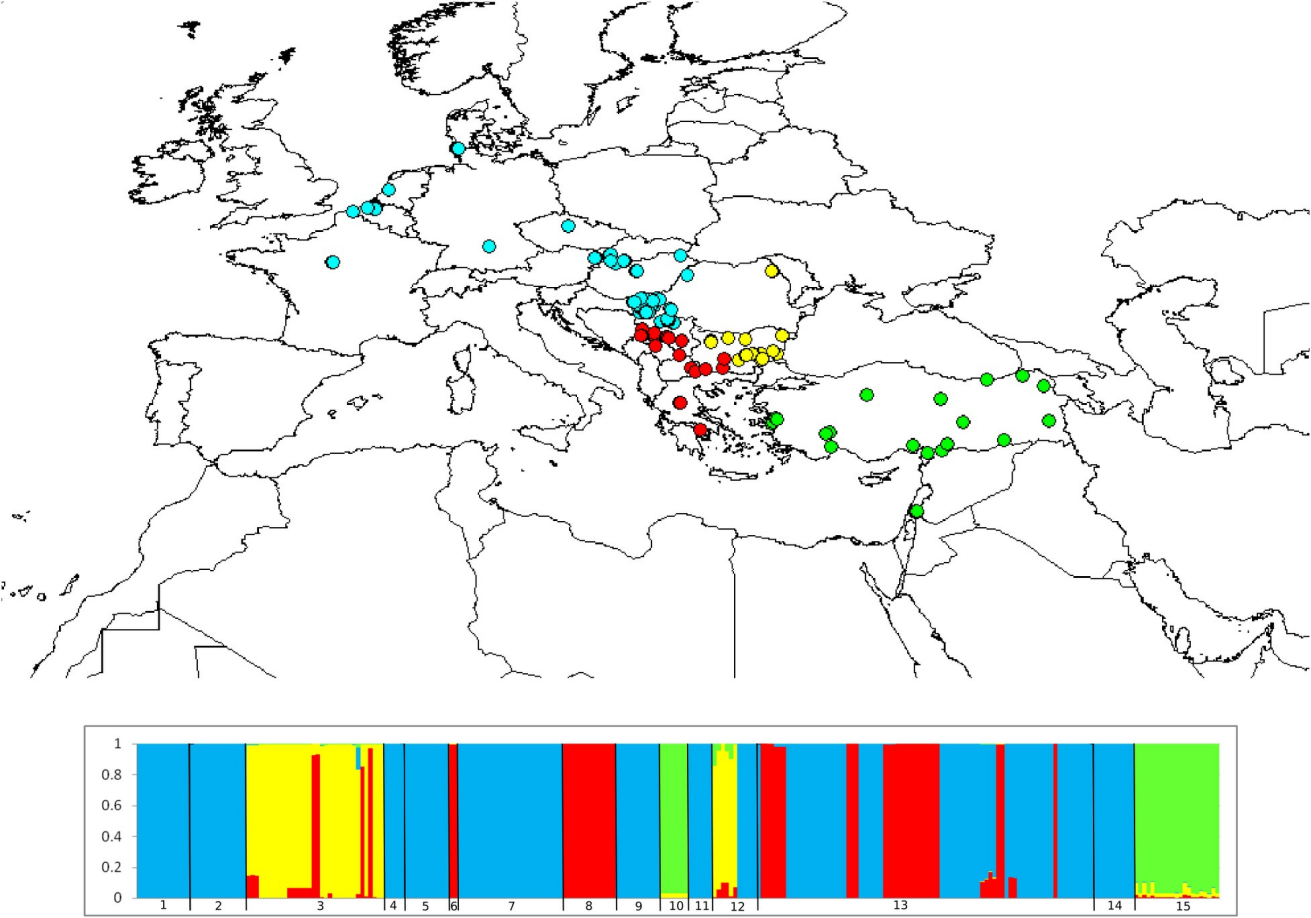
Map of spatial cluster memberships of *L*. *europaeus* individuals from Europe and Anatolia/Middle East according to their *MT-ND6* sequences and bar plot with probabilities for membership to each of the clusters (1-Austria; 2-Belgium, 3-Bulgaria, 4-Czech Republic, 5-France, 6-North Macedonia, 7-Germany, 8-Greece, 9-Hungary, 10-Israel, 11-Netherlands, 12-Romania, 13-Serbia, 14-Slovakia, 15-Turkey). Circles represents the individual sampling localities, while the circles colour represent cluster membership (blue for CE–central Europe; red for CB—south and central Balkans; yellow for EB–southeastern and eastern Balkans; green for AME–Anatolia/Middle East).

The Geneland analysis revealed the following four spatial groups: central Europe (CE), south and central Balkans (CB), southeastern and eastern Balkans (EB) and Anatolia/Middle East (AME) (Fig 3). That spatial differentiation was further supported by significant pairwise Φ_ST_ values (Table 2) and our AMOVA (Φ_ST_ = 0.559; p < 0.001) that indicated that most of the genetic variability (55.97%) was due to differentiation among individuals between the spatial groups. The highest molecular diversity was found within the AME spatial group, whereas hares of the EU spatial group showed the lowest diversity values (see Table 3 for molecular diversity indices of the four spatial groups).

**Table 2:** Pairwise Φ_ST_ values between the four spatial genetic groups detected in the brown hares from Europe and Anatolia/Middle East.

	CE	CB	AME
CE			
CB	0.554		
AME	0.818	0.204	
EB	0.263	0.411	0.707

**Table 3:** Molecular diversity indices and neutrality tests in the four detected spatial genetic groups of brown hares from Europe and Anatolia/Middle East. CE–central Europe; CB—south and central Balkans; EB–southeastern and eastern Balkans; AME–Anatolia/Middle East.

	CE	CB	EB	AME	Total
Sample size	158	45	36	28	267
Number of haplotypes	20	13	14	18	58
Haplotype diversity (Hd)	0.573	0.851	0.889	0.960	0.817
Nucleotide diversity (π)	0.002	0.004	0.014	0.008	0.008
Average number of pairwise differences (k)	0.962	2.032	7.205	4.251	4.055
**Neutrality tests**					
Tajima D	-2.316**	-0.996	0.516	-0.884	-1.760*
Fu’s Fs	-4.196*	-1.923	0.509	-0.584	-2.086
Mismatch distribution	0.0001	0.014*	0.042*	0.003	0.015

**p<0.01

*p<0.05

Our MJ Network and spatial clustering of individuals in Geneland revealed by and large the pattern as obtained for the D-loop sequences [21], suggesting that both of the examined markers followed the overall neutral expectations of gene flow from Anatolia/Middle East to the Balkans, and furthermore to central Europe, with gradual decrease in molecular diversity indices from south to north. Our BPEC analysis further supported the proposed phylogeographic partitioning, marking the locations of northeastern Anatolia as the most ancestral, similar to the root of the MJ network connecting to the ancestral (AM) haplotype H23 from the Moldova region in Romania.

Among the 56 polymorphic sites, the total number of synonymous changes was 48, while 14 sites were nonsynonymous substitutions translating into 17 different amino acid sequences i.e., protein variants, named as A to Q (Fig 1, S1 Fig, Table 1). Protein variant A was the most frequent (65.17%) among all variants recovered; it was also the most widely distributed variant among all variants in Europe. Whereas this protein variant did occur exclusively in Europe, protein variant B with an overall frequency of 12.35% was mostly distributed in Anatolia and Israel. It was the most ancestral variant according to our rooted MJ network, and all other protein variants were eventually derived from it. We never observed more than one nonsynonymous substitution between any two evolutionarily neighboring protein variants; even not between the protein variants B and A; the latter differed only at one nonsynonymous position (I126V) from protein variant B. However, protein variant A differed from variant B by additional five synonymous substitutions between the two closest haploptypes (one unsampled haplotype of the protein variant B and H1 of the protein variant A); four of those five synonymous substitutions had nucleotides unique for haplotype sequences of either the B or the A protein variants, respectively. Thus, given that the protein variant B was considered as the most ancestral variant, those four synonymous nucleotide positions represented synonymous mutations within the B protein variant. The one remaining synonymous substitution could not unambiguously be assigned to the protein variant B or A.

Even though nucleotide sequence diversity (Hd, π) was higher in the AM phylogroup than in the EU phylogroup, the number of protein variants relative to all substitutions per each phylogroup was around four times lower (p < 0.05, d.f. = 2, Fischer Exact test) in the AM phylogroup (% of nonsynonymous substitutions = 7.69) than in the evolutionarily younger EU phylogroup (% nonsynonymous substitutions = 30.56).

In order to detect codons under positive selection, we performed three likelihood ratio tests, comparing M0 vs. M3, M1a vs. M2a and M7 vs. M8. A significant difference between the fit of model M0 vs. M3 was found (2ΔLnl = 93.0168, d.f. = 4, p < 0.001), as well as when comparing the models M7 vs. M8 (2ΔLnl = 21.92, d.f. = 2, p<0.001), whereas no significant difference was found when comparing models M1 vs. M2 (2ΔLnl = 6.03, d.f. = 2, p = 0.049). Following the Bayes Empirical Bayes analysis in the comparison of M7 vs. M8 three codons were indicated to be under positive selection, namely codon positions 4, 102, and 126, with posterior probabilities higher than 95%. No evidence of positive selection was confirmed by SLAC and MEME tests provided by web server Datamonkey, while FEL analysis showed only codon position 102 to be affected by positive selection (p = 0.085). Furthermore, the REL analysis supported the positive selection at three codon positions: 4, 92 and 102. Thus, having in mind all performed approaches in PAML and Datamonkey web server, only codon position 102 was confirmed by more than two tests. This codon position contains three possible different amino acids defining three proteins/protein groups: 1) Methionine leads to proteins D, M, and J distributed in Bulgaria and southern Serbia; 2) valin leads to protein F, occurring in Bulgaria; 3) and leucine leads to the widely distributed protein variant A, as well as E, H, I, K, L, N, O,P, Q (for their distribution see Figs 1 and 2). The prediction of the secondary structure showed that codon 102 was located within the transmembrane helix number 4. Codon position 4 was proved under positive selection by two likelihood approaches, separating the protein variants E and N (occurring in Serbia and Hungary) with leucine at this position from protein C with valine (occurring almost exclusively in Bulgaria; only in one individual from Turkey), while all other variants had methionine at this position. Our secondary structure prediction places this position at the beginning of the N-terminus within the alfa helix of the transmembrane domain number 1 of the *MT-ND6* protein.

Our multinomial regression models to test for significant climatic effects on the occurrence of protein variants as resulting from the positively selected position were restricted to samples from Europe, because in Anatolia and Israel only two individuals had other proteins than the protein variant B and only one among those two individuals harbored protein variant G that differed from B by one substitution under positive selection proved only by the REL approach. Moreover, samples from Austria, Germany, Belgium, the Netherlands, and France were not included in the models, as these regions harbored exclusively the most prevalent and ancestral European protein variant A; supposedly, all other protein variants occurring in the Balkans at low frequencies simply did not have the chance of migrating into central Europe that has been colonized very recently in evolutionary terms (i.e., after the late glacial maximum, see also [21]). Furthermore, our multinomial regression models were based on only six protein variants (A, B, C, D, G, F) as dependent variable, as they occurred at the minimum sample size (n = 6) found for any protein variant (F) resulting from a positively selected codon position. In total, the modeling was based on 150 individuals. Our non-rotated PCA of the temperature data for the individual collection localities (see Material and methods) was based on a correlation matrix and extracted two principal components that together explained 91.97% of the original variable variance (the first temperature component explained 77.5% and the second 14.47%). Based on the respective loadings of the original variables, the first temperature component (temperature factor 1) could be interpreted as a general temperature component except for the wettest quarter of the year, whereas the second temperature component (temperature factor 2) specifically reflected the temperature of the wettest period of the year. The non-rotated PCA of the precipitation data was also based on a correlation matrix and extracted two principal components that explained 44.81% (precipitation factor 1) and 43.04% (precipitation factor 2) of the original variable variance, respectively. Based on the respective variable loadings the precipitation factor 1 could be summarized as a general precipitation factor that accounted specifically for precipitation during the coldest and wettest season of the year. The precipitation factor 2 could be interpreted as precipitation during the driest and warmest period of the year. However, due to serious multicollinearity problems (as already indicated by linear Pearson correlation coefficients with latitude and precipitation factor 2: below -0.6, respectively), both temperature factors were excluded from our multinomial regression models. Thus, our full multinomial regression model of protein variant occurrences included the following controlling variables: longitude, latitude, altitude, precipitation factor 1, and precipitation factor 2. Based on the Akaiki criterion corrected for small sample sizes (AICc), the best model (AICc = 238.68; delta = 0) was the one including the controlling variables longitude, latitude, precipitation factor 1, and precipitation factor 2. The second best model (AICc = 245.43; delta = 6.75) included also altitude in addition to all those variables that were included in the best model. The best model indicated significant effects of the precipitation factor 1 (regr. coeff. = - 1.599, p = 0.009) and precipitation factor 2 (regr. coeff. = 2.294, p = 0.016) on the occurrence of protein variant D, when compared to the occurrence of protein variant A. Also, the occurrence of protein variant F was significantly affected by the precipitation factor 2 (regr. coeff. = 6. 230; p = 0.032) in the best model as compared to protein variant A. Moreover, there was a significant latitudinal effect on the occurrence of protein variant D (regr. coeff. = -1.396, p = 0.001) and a longitudinal effect on protein variant F (regr. coeff. = 1.162, p = 0.004), as compared to protein variant A. Other protein variants exhibited only significant geographical differences as compared to protein variant A (for variant B a longitudinal effect: regr. coeff. = 0.832, p = 0.008; and for variant C a latitudinal effect: 2.396, p = 0.005).

## Discussion

In our study we detected positive selection acting on the mitochondrial *NADH* subunit 6 (*MT-ND6*) gene of brown hares (*Lepus europaeus*) from different biogeographic regions with different regional evolutionary history. In fact, positive selection on diverse mt OXPHOS genes has been proved in a spectrum of animal species in various ecological contexts over almost the last two decenia. For instance, Balloux et al. [50] reported a significant effect of ambient temperature on the variation of human mtDNA and Consuegra et al. [1] suggested adaptation of mtDNA in Atlantic Salmon to higher metabolic efficiency at lower ambient temperature. Foote et al. [51] observed positive selection and associated radical physico-chemical property changes in mt proteins of killer whales, and Garvin et al. [52] found positive selection of mt OXPHOS genes of Pacific salmons. Recently, Melo-Ferreira et al. [10] investigated total mitogenomes of eleven hare (*Lepus*) species and pointed out that hares may represent an ideal evolutionary group to study adaptation processes of mt genes (that are maternally inherited and not recombining, with very few exceptions), as they represent an evolutionarily young radiation with adaptation to many different environments and climates; they also may show signatures of reticulate evolution regionally, such as introgressive hybridization or shared ancestral polymorphism due to incomplete lineage sorting because of their mode of shallow evolutionary divergence. Evolutionary patterns of selection of some few mt OXPHOS genes across a number of hare species have been studied and also in the context of potential adaptation to climate, and specifically adaptation to cold environments [6, 10]. Temperature and precipitation effects on the occurrence of protein variants of the *MT-ATP6* and *MT-ND2* genes have been found in a population genetic study on cape hares, *Lepus capensis* sensu lato, across a steep ecological gradient within a relatively short geographic distance in north Africa despite high gene flow in the nuclear genes as indicated by microsatellite variation [7].

Indeed, given the functional importance of mt OXPHOS genes for cellular energy production and the fact that single mutations can have serious (negative) effects and may result in mitochondrial disorders and diseases (e.g., [15–16,53–54]), we expected positive selection on those genes particularly under spatial and temporal heterogeneity in the environment. Even though the origin of adaptations in mtDNA remains elusive, positive selection acting on the OXPHOS genes has been interpreted as an adaptation to varying environmental characteristics, and in the absence of experimental studies “correlative” (i.e., statistical) effects of environmental factors such as climatic parameters on the (spatial) occurrence of mt OXPHOS protein variants are at least being considered as indicative of adaptation.

In the present study, we examined the effect of selection on amino acid sequence variants of the *MT-ND6* and adaptation signals in brown hares from Europe and Asia Minor with special emphasis on the phylogeographic history of this species. Brown hares are one among many mammal species with temporal distribution that has been strongly influenced in their past distribution by the climatic perturbations and concomitant habitat/vegetation changes during the Late Pleistocene. Despite their obvious ability to establish thriving populations in a wide range of ecological habitats–currently they are present in many regions of temperate, mediterranean, semiarid, tropical, subarctic/subantarctic environments [19, 55], they were absent from large parts of temperate continental Europe during the Late Pleistocene due to extensive ice shields or too harsh arctic tundra conditions. Rather, their distribution was restricted mainly to the (central and southern) Balkans in the southeast of Europe or to the southwest of Eurasia (e.g., the Crimean Peninsula) with less harsh (winter) climate. Several phylogeographic studies based on mitochondrial D-loop sequences indicated a rather continuous evolutionary development of lineages from Anatolia Peninsula into the southeastern Balkans. The latter has acted as a source region for further colonization of the Balkan Peninsula and later on–after the last glacial maximum (ca. 18.000 ybp)–to central Europe [20–22, 24]. Particularly the study of Djan et al. [21] also suggested a long lasting ancestral population history in the Anatolian Peninsula [29], a subsequent population expansions in the southeastern and southern Balkans, and a relative young population expansion into central and northern/northwestern continental Europe. Additional population expansion in eastern Europe, probably from southwestern Eurasian regions with lineages phylogenetically closely related to those found on the Anatolian Peninsula and other regions in the Near East were particularly suggested by Ashrafzadeh et al. [20]. Those latter mentioned lineages have, however, so far not managed to expand much towards central Europe; rather their western front does not seem to extend much further west than eastern Poland and the Ukraine. Whether that eastern mitochondrial expansion has already substantially reached Moldova and the eastern parts of Romania needs to be investigated, and a comprehensive mitochondrial phylogeographic study of Bulgarian brown hares [56] including hares from the larger geographic context is also pending. These data suggest ancient gene flow into the Balkans exclusively from the Anatolian Peninsula rather than (substantial) gene flow from north and northeast of Bulgaria (i.e., from north of the Black Sea). Nevertheless, D-loop sequence diversity in the southeastern Balkans, i.e., Bulgaria as well as Greece and Turkish Thrace, is relatively high, due to the presence of Anatolian lineages in addition to the typical “European”lineages that are all phylogenetically younger than the Anatolian ones and that are entirely absent from the Anatolian Peninsula. In spite of the presence of both Anatolian and typical “European”lineages in the southeastern and southern Balkans, D-loop sequence diversity of brown hares from the Anatolian Peninsula is still higher than in the Balkans. This pattern of diversity is also reflected by the nuclear gene pool as evidenced by both allozyme and microsatellite data [28, 32].

Even though occasional heteroplasmy, shared ancestral polymorphism, and evolutionary processes such as recombination resulting from positive selection on various combinations of amino acid sequences at certain mt OXPHOS loci could lead to incongruence of phylogeographic patterns as obtained by different mtDNA genome fragments, our study indicated that the *MT-ND6* phylogeographic pattern followed by large the model based on the neutrally evolving mtDNA D-loop sequences published by Djan et al. [21]. This is not astonishing as the overall mt phylogeographic pattern is derived by far from neutrally evolving positions and only one positively and some negatively selected sites. Thus, the geographical distribution of the main two groups of *MT-ND6* protein variants can be understood as having resulted from those historical events during the (late) Pleistocene as described above. However, given the different mutation rates and constraints in mtDNA for different nucleotide sequences, the phylogenetic gap between the haplotypes of the Anatolian Peninsula and the Balkans haplotypes as identified earlier by D-loop sequences [21, 22, 24] has almost disappeared for the currently analyzed *MT-ND6* sequences: only six mutations do separate the two *MT-ND6* phylogroups “Anatolia/Middle East”and “Europe“, with only one nonsynonymous substitution; and this is in line with the interpretation of continuous phylogenetic evolution of the mitogenomes of brown hares from the Anatolian Peninsula/Middle East and brown hares from Europe–it indicates direct evolution of the most ancestral *MT-ND6* protein B from the Anatolian Peninsula into the most ancestral and widespread European protein variant A via one nonsynonymous position (I126V). Seemingly, the currently observed geographic distribution of all discovered *MT-ND6* protein variants is essentially reflecting the earlier reported overall (neutral) phylogeographic pattern. In parallel to that “neutral phylogeographic scenario“, the currently observed nucleotide diversity of our *MT-ND6* sequences is significantly higher in the (more ancestral) haplotypes from the Anatolian Peninsula and the Middle East than in Europe. However, contrary to that neutral expectation, the diversity of the currently observed *MT-ND6* protein variants is significantly higher in the evolutionarily younger “European haplotypes”than in the typical “Anatolian/Middle Eastern haplotypes“, and it is particularly rich in the lineages from the Balkans. Whereas only two nonsynonymous positions (M4V, L92F) and correspondingly three protein variants (B, C, G) were found among all ancestral lineages of the “Anatolian/Middle Eastern”phylogroup, a total of eleven nonsynonymous substitutions (M4L, V5M, M11V, I86V, M91V, L92F, V93A, L102M, L102V, M102V, A107V, V108M, G133D) and correspondingly fourteen protein variants (A, D, E, F, H, I, J, K, L, M, N, O, P, Q) were recovered within the evolutionarily younger European phylogroup. Moreover, among those nonsynonymous positions of the evolutionarily younger phylogroup in Europe, one position (L102M, L102V, M102V) was proved under positive selection. Notably, that positively selected site did not occur in an evolutionarily ancestral “Anatolian lineage”also occurring in the Balkans, but along a young (short) evolutionarily pathway derived from the most ancestral European protein. North of the Balkans the diversity of *MT-ND6* protein variants is markedly lower, presumably because of the short evolutionary period of time after the late glacial maximum (less than ca. 20.000 generations) for mutations and/or for immigration of less frequent protein variants from the Balkans into central Europe. This interpretation is in parallel with nucleotide diversity patterns as observed from the neutral D-loop sequences [21, 22, 24]. For the “Anatolian/Middle Eastern haplogroup”negative selection at some sites (e.g., codons 28, 101, 103, 167) may specifically have led to more preservation within the protein system B of the brown hares from the Anatolian Peninsula and the Middle East. It is conceivable that the evolutionarily more recent (late Pleistocene) and severe climatic perturbations in Europe that have culminated in dramatic climatic and vegetation changes during the last glacial maximum could have imposed stronger selective pressures on the evolution of the “protein A variant group”that is exclusively distributed in Europe, and that could have lead to the higher protein diversity currently observed in Europe. In fact, terrestrial pollen records from central and eastern Mediterranean areas indicated that those regions were highly sensitive to vegetation changes during the Pleistocene, in contrast to sedimentary records from the entire Near East where abrupt climate oscillations were rare [57]. The European protein variants D, F, and E have evolved directly by mutations at the codon position 102 that has been proved under positive selection. Moreover, the occurrence of the protein variant D was significantly affected by precipitation as compared to the most ancestral and most common European variant A. In particular, protein variant D was favored at locations of lower general precipitation values during the wettest and coldest period of the year, or of higher precipitation during the driest and warmer period of the year. Similarly, protein variant F was favored at locations of high precipitation during the warmest and driest period of the year. Altitudinal diversity leading to larger variation of precipitation across the Balkans may have imposed particularly during late periods of the Late Pleistocene (e.g., the late glacial maximum some 18.000 ybp) and thus have affected the distribution of the MT-ND6 variants in that part of Europe.

Based on the analyses of complete mitogenomes of eleven hare species Melo-Ferreira et al. [10] found only two codon positions within *MT-ND6* (14 and 108) to be under positive selection, as suggested by at least two different methods, but none of those positions was suggested by our selection tests, and on the contrary the position that we found under positive selection (102) was not identified under positive selection by Melo-Ferreira et al. [10]. We think that both the number of different haplotypes studied, their evolutionary divergence, and the overall sample composition may affect the outcomes of selection tests. Our secondary structure predictions placed all of the amino-acid positions exhibiting a signal of positive selection within the transmembrane helical domains of the *MT-ND6* protein, similar to the study of Yu et al. [17] where two positions under positive selection of *MT-ND2* and *MT-ND6* sequences of snub-nosed monkeys from China were also proved to be located within the transmembrane domains, while the position of *MT-ND6* was found to be within the same transmembrane domain as codon position 102 revealed in our study, but also as position 108 revealed in Melo-Ferreira et al. [10]. Contrary, in a study of adaptive evolution in mammalian mitochondrial genomes da Fonseca et al. [8] suggested that signals of adaptive variation in the entire NADH dehydrogenase complex have been almost solely confided to the loop regions.

In conclusion, we observed a signal of positive selection on the *MT-ND6* gene of brown hares from Europe, specifically the Balkans, where phylogeographically relatively young sequences prevailed compared to the relatively ancestral lineages from the Anatolian Peninsula and parts of the Middle East that are phylogeographically the only sources of the Balkan lineages. No such positive selection signal was observed in the lineages from the Anatolian Peninsula, despite their higher sequence (nucleotide) diversity and older evolutionary age. In parallel, although evolutionarily younger than the lineages from the Anatolian Peninsula, the European lineages exhibited higher diversity of protein variants and fewer positions under negative selection than the lineages from the Anatolian Peninsula. Moreover, the occurrence of protein variants on the Balkans that were derived through the positively selected position was significantly affected by precipitation variation in the Balkans. That latter finding was interpreted as signal of adaptation to (regionally and/or temporally) varying climate regimes probably during periods of the late Pleistocene when climatic perturbations were more severe in (southeastern) Europe than in the Anatolian Peninsula and parts of the Middle East.

## Supporting information

S1 Fig Geographic positions of sampling localities of *L. europaeus* from Europe and the Middle East, together with the geographic distribution of all detected protein variants shown as corresponding letters of protein designation. (PDF)
