# Supplementary figures and images for: Positive selection and precipitation effects on the mitochondrial NADH dehydrogenase subunit 6 gene in brown hares (Lepus europaeus) under a phylogeographic perspective
Source: PLoS One. 2019 Nov 8;14(11):e0224902. doi: 10.1371/journal.pone.0224902 (PMC6839855; doi:10.1371/journal.pone.0224902)

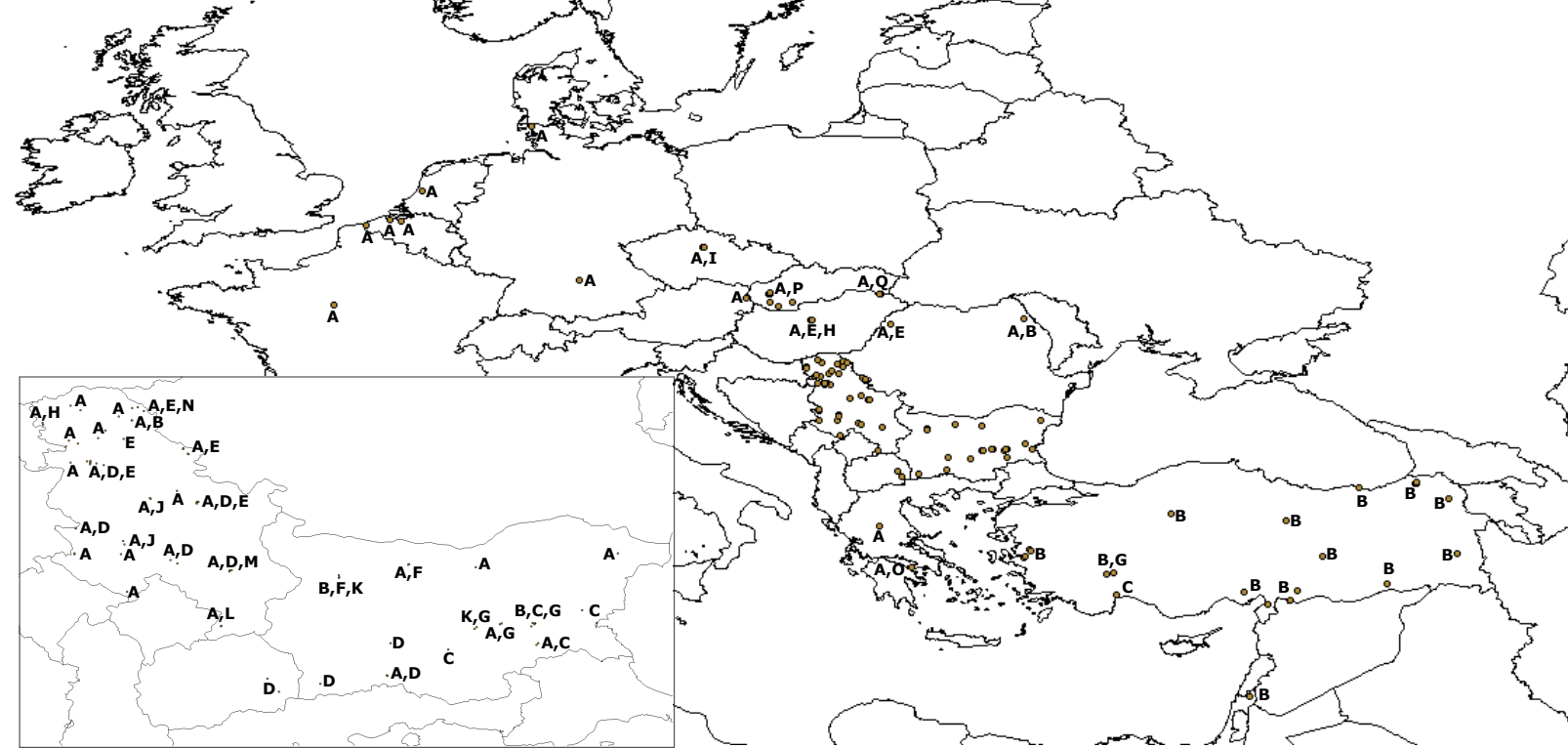

Supplement: S1 Fig — (PDF) [file pone.0224902.s001.pdf]
